# Uterus Transplantation: From a Deceased Donor or Living Donor?

**DOI:** 10.3390/jcm11164840

**Published:** 2022-08-18

**Authors:** Iori Kisu, Kouji Banno

**Affiliations:** Department of Obstetrics and Gynecology, Keio University School of Medicine, 35 Shinanomachi, Shinjuku-ku, Tokyo 160-8582, Japan

Uterus transplantation (UTx) is a new alternative to surrogacy or adaption for women with uterine factor infertility to have a child. It was built after great achievements in basic research with animal experiments, including non-human primates, accumulated over a decade [1,2,3]. While UTx is still at the clinical experimental stage, this new reproductive and organ transplant technology is spreading rapidly worldwide, mainly in Europe and the USA, as well as Asia [4,5]. To date, more than 80 UTx procedures have been performed, resulting in more than 40 live births from deceased or living donors in multiple centers. With the proof of concept of UTx as a new treatment for uterine factor infertility, the International Society of Uterus Transplantation (ISUTx), founded in 2016, was officially accredited as the 10th official section of organ transplant in the Transplantation Society in 2021.

In the procurement of uterine grafts, potential origins have been developed with living donors, deceased donors, or both [6,7], depending on center policies. To date, approximately two-thirds of UTx procedures have been performed on living donors. Regarding the surgical success rate of the UTx procedure, it is slightly higher in UTx with a living donor (LD) (78%) than with a deceased donor (DD) (64%) [4]. However, in a recent cohort study from three centers in the USA, the overall 1-year graft survival rate was 74%, including 74% after LD UTx and 75% after DD UTx, which shows that each procedure is comparable [8]. Considering the potential origins of uterine grafts, whether using deceased or living donors, it is necessary to fully understand the advantages and disadvantages of these procedures [9,10,11].

The world’s first surgical success of UTx, with long-term graft survival and spontaneous menstruation, was performed by a team from Turkey in 2011, which was conducted on a DD [12]. The patient had five repeated miscarriages after UTx surgery but eventually led to birth 9 years after UTx [13]. The first successful live birth following UTx from a DD was achieved by a team in Brazil in 2017 [14]. In addition to these teams, live births following UTx with DD have been reported in some places, including Cleveland, Dallas, the Czech Republic, and Pennsylvania [15].

The guiding principles of organ transplantation still indicate that the organ should be procured from a DD in view of the greatest advantage of eliminating the surgical burden of LDs. In addition, resection of extended vascular pedicles of greater length than those from LDs with the uterus can be achieved with a faster procurement time. Moreover, donor candidates can potentially be younger than DDs [11,16]. However, UTx from a DD also has the following disadvantages: (i) Less comprehensive assessment. Careful preoperative preparation, thorough preoperative screening, adequate informed consent, and medical and obstetrical histories are limited because of the inability to plan the surgery. (ii) Suboptimal patient selection and shortage of deceased donors. Potential DDs are restricted and in shortage depending on the country’s situation. Therefore, ideal donors who fulfilled the inclusion criteria, including age, body mass index, smoking, past medical or surgical history, and HLA matching, could not be enrolled [11]. (iii) Longer ischemic time. The ischemic time in UTx from DDs was longer than that from LDs. Specifically, the cold ischemia time is much longer than that in LDs, because the procured uterus is transferred to another institution. Furthermore, the uterus of a brain-dead multiorgan donor is not a vital organ and it is the final procurement of vital organs, resulting in an extended ischemic time. These factors could affect the viability and functionality of the graft because of ischemic–reperfusion injury. The maximum allowable ischemic time of the uterus remains undetermined and the optimal composition of the graft-storage medium remains unknown for this organ [17,18]. (iv) Influence of the agonal stage on the graft. Vasoactive drugs, including catecholamines, are frequently administered to patients in the agonal stage. Consequently, negative effects on organs may be caused by vascular insufficiency and systemic inflammatory changes. No UTx procedures have been performed on a donor who died from cardiac disease and this is controversial. (v) Need for delicate back-table preparation. In uterine procurement from an LD, microvessels located in tissues surrounding the uterus can be carefully ligated during LD surgery, whereas in DD surgery, vessel dissection and ligation are mainly performed on the back table. Because it is difficult to find fine vessels around a white uterus that has been washed out with an organ-protecting solution, inadequate preparation may result in hemorrhage after reperfusion during recipient surgery [14]. Despite the above disadvantages of DD UTx, none outweigh the greatest benefit of avoiding any burden on the LD. Unfortunately, there are countries, such as Japan, where the uterus is not legally included as a transplant organ and, therefore, cannot be implemented [19].

Regarding UTx from an LD, the first UTx procedure was conducted in 2000 in Saudi Arabia, resulting in a surgically unsuccessful case with perioperative unilateral laceration of the donor ureter [20]. After accumulating data from animal experiments, the first surgical success of LD UTx was achieved by a Swedish team in 2012, leading to the world’s first successful live birth after UTx in September 2014 [21]. This remarkable achievement has provided great hope for couples with no children because of uterine factor infertility. In the wake of this success, the clinical application of UTx has rapidly spread worldwide.

The ultimate advantage of LD UTx is that the surgery can be scheduled and the candidate can be enrolled after a thorough physical and psychological evaluation with details of medical and obstetrical history. UTx between relatives (especially from mother to daughter) is most likely to be performed [7,16,22,23] and human leukocyte antigen (HLA) matching is more likely. However, they could be less-than-optimal candidates because of the advanced age of the mother. By contrast, in the USA, a living-organ donation is not restricted to recipients’ relatives, resulting in more potential LDs being available [6].

The most serious problem with LD UTx is that donor surgery is highly invasive, has a long operative time, and is extremely complicated. Moreover, the rate of postoperative complications requiring surgical, endoscopic, or radiological interventions among LDs is high (18%) and the majority of these complications are urinary tract injuries [4]. Thus, an extremely precise surgical procedure is likely required to dissect the deep uterine and internal iliac veins located in the pelvic floor as drainage veins, preserving the vesical nerve branches of the hypogastric nerve and inferior hypogastric plexus around the deep uterine vein to prevent postoperative dysuria [24]. Recently, a surgical procedure using the utero-ovarian vein or ovarian vein instead of the deep uterine vein was introduced as a drainage vein by multiple centers to reduce surgical invasion of an LD [25]. However, the use of the ovarian vein from premenopausal women is controversial because the procedure requires oophorectomy, which may cause ovarian deficiency symptoms and necessitate hormone-replacement therapy to prevent the risk of cardiovascular disease and osteoporosis. In the introduction stage of UTx, laparotomy was mainly used, but robot-assisted donor surgery is currently becoming mainstream to reduce the risk of LDs [23]. Regarding other risks in LD, the mental burden of invasive surgery, familial pressure to donate (voluntariness of consent), decreased quality of life due to complications of hysterectomy, and sexual dysfunction should be considered.

Whether from LD or DDs, for UTx, each procedure has advantages and disadvantages. Each center is expected to establish the proof of concept and feasibility of UTx with the most appropriate means after fully considering these characteristics. We hope that UTx will become a notable milestone in the field of reproductive medicine and organ transplantation, with the accumulation of scientific evidence.
